# Trismus Nascentium Successfully Treated with Sulphate of Quinine

**Published:** 1860-03

**Authors:** H. A. Johnson

**Affiliations:** Chicago


### TRISMUS NASCENTIUM SUCCESSFULLY TREATED WITH SULPHATE OF QUININE.

By H. A. JOHNSON, M. D., Chicago.

I was called Dec. 25th, 1859, to see the infant child of Mrs C., which had been seized about two hours previously with convulsions. The child was a female, two days old, and up to the time of the attack apparently in perfect health.

The convulsions were tetanic in their character, and accorded perfectly with the symptoms of this disease as described by those who have seen it frequently in more Southern latitudes. Two similar cases occurring in my own practice both died.

The head was thrown back; the breathing difficult, and at times almost entirely suspended; the skin was moderately cool, and the pupils quite natural in appearance. The heart's action during the paroxysms which recurred at intervals of from twenty to fifty minutes, was very much reduced in frequency, apparently on account of the asphyxia. The jaws were closed, with partial and sometimes complete relaxation betweeu the paroxysms.

Previous to my arrival the child had been placed in a warm bath, without any perceptible benefit. The bowels were immediately moved by an enema, and ipecac given *ad nauseam.* Sinapisms were applied to the extremities. The kidneys were acting normally. Subsequently Fl. Ext. Conii was given internally.

None of these means, so far as I could judge, had any beneficial influence. The slightest noise in the room, a jar or even a ray of light, was sufficient to induce severe general spasms. At 11 o'clock in the evening of the second day, thirty-four hours from the first attack, I gave the little patient one grain of sulphate of quinine. In a few minutes a severe spasm came on, differing in no respect from the fifty or sixty that had preceded it. The next interval was a full hour, a longer period than any which had hitherto occurred. The paroxysm was slight and shorter in duration than usual. For the next two hours the patient rested in a sweet and gentle sleep. On awaking there was a little closure of the jaws, and slight spasm of the muscles of respiration, but nothing in comparison to the fearful contortions that we had previously witnessed. For the next five hours there was not the first symptom of a convulsion but at 7, A. M. the lips became somewhat livid and the breathing irregular. This lasted only for a few minutes. I immediately gave another grain of the sulphate of quinine. From that time there has been no symptoms of a return of the disease, and the child is now perfectly healthy.

*Remarks.*—In the first number of the *Examiner*, my colleague, Prof. Andrews, reports a case of tetanus treated by me some years since with large doses of sulph. quinine. The patient recovered. I was induced to resort to it in that case from having seen, I think in the *New Orleans Medical Journal*, the report of a case successfully treated by this agent. The analogy between traumatic tetanus and the convulsions occurring in new born children, led me to the use of the same remedy in the case detailed above.

The points of special interest in the case are:

*First.*—The extreme rarity of the disease in this latitude. Old practitioners claim that it is never met with here.

*Second.*—The locality in which this case occurred was one of the most healthy in the city, near the lake shore, where there was always an abundance of pure bracing air. The mercury at the time was but a few degrees above zero.

*Third.*—The mother previous to her confinement had been in perfect health and the child had been properly cared for; there was no inflammation or ulceration about the cord; the bowels and kidneys were normal in their action.

*Fourth.*—The chief point of interest in the case consists in the fact, that while the disease is almost uniformly fatal under whatever treatment has hitherto been adopted, this little patient after having suffered continuously for thirty-four hours was effectually and permanently relieved after the administration of two doses of sulph. quinine of a grain each, the relief beginning to be experienced within an hour after the first portion.

A grain of the sulph. quinine is certainly a large dose for an infant three days old, but if given at all in such cases I think the quantity should be sufficiently large to produce its full sedative effect upon the nervous system. In this case I fully believe that it saved the life of my patient.

